# Machine‐Learning Prediction of Bleeding After Endoscopic Submucosal Dissection for Early Gastric Cancer: A Multicenter Study

**DOI:** 10.1002/jgh3.70203

**Published:** 2025-06-29

**Authors:** Hiroki Maruyama, Kazuya Takahashi, Kosuke Kojima, Nao Nakajima, Hiroki Sato, Ken‐ichi Mizuno, Soichi Sugitani, Shuji Terai

**Affiliations:** ^1^ Division of Gastroenterology & Hepatology Graduate School of Medical and Dental Sciences, Niigata University Niigata Japan; ^2^ Division of Gastroenterology Koseiren Murakami General Hospital Niigata Japan

**Keywords:** artificial intelligence, bleeding, early gastric cancer, endoscopic submucosal dissection, feature importance, machine learning, Shapley aAdditive explanations (SHAP)

## Abstract

**Background:**

Endoscopic submucosal dissection (ESD) is a minimally invasive treatment for early gastric cancer (GC); however, post‐ESD bleeding remains a serious and unpredictable complication. This study aimed to develop machine‐learning (ML) models to predict post‐ESD bleeding and identify associated risk factors, ensuring accurate and interpretable predictions.

**Methods:**

A retrospective, multicenter clinical database was constructed for patients who underwent ESD for early GC. An ML model was developed using patient characteristics and perioperative findings to predict bleeding within 28 days post‐ESD. Its performance was compared with that of a logistic regression–based non‐ML model. Feature importance analysis was performed to aid interpretation.

**Results:**

Among 1084 patients (median age: 75 years), post‐ESD bleeding occurred in 63 (5.8%). The area under the curve of the ML model was better than that of the non‐ML model (0.80 vs. 0.71, *p* = 0.03). Furthermore, the ML model demonstrated a trend toward higher sensitivity compared with the non‐ML model (0.74 vs. 0.58, *p* = 0.58). When stratified by ML‐estimated bleeding probability, observed bleeding rates were 2.3%, 8.8%, and 28.6% in the low‐ (< 50%), intermediate‐ (50%–80%), and high‐probability (≥ 80%) groups, respectively. The odds of bleeding were significantly higher in the intermediate‐ (OR 4.03, *p* = 0.03) and high‐probability (OR 16.7, *p* < 0.01) groups compared to the low‐probability group. Anticoagulant use with atrial fibrillation emerged as a key predictor.

**Conclusions:**

The ML model effectively rules out post‐ESD bleeding and identifies clinically meaningful risk factors, supporting its use in personalized prophylactic strategies.

## 1 Introduction

Gastric cancer (GC) remains the fifth most common cancer and the fourth leading cause of cancer‐related death globally [1]. Advanced GC requires intensive treatment, including surgery; however, local endoscopic treatment can be curative when detected early due to the minimal risk of lymph node metastasis [2]. Endoscopic submucosal dissection (ESD) has become the standard minimally invasive treatment for early GC without lymph node metastasis and is widely performed worldwide [3]. Although ESD is less invasive than gastrectomy, post‐ESD bleeding remains a major complication, with an incidence ranging from 3.1%–6.5% [4, 5, 6]. Post‐ESD bleeding can lead to serious clinical outcomes, including prolonged hospital stay, deterioration of nutritional status, and death from hemorrhagic shock in severe cases [7, 8]. Therefore, if post‐ESD bleeding can be predicted, perioperative risk management can be implemented proactively, thereby enhancing the safety of ESD.

To date, various risk factors for post‐ESD bleeding have been reported, including the use of antithrombotic agents such as anticoagulants and P2Y12 receptor antagonists (P2Y12RA), heparin bridging, hemodialysis, hypertension, and large resection size [9, 10, 11]. With the aging global population, the prevalence of comorbidities, such as hypertension or ischemic heart disease/atrial fibrillation (Af) requiring antithrombotic agents, is expected to rise [12, 13, 14, 15]. As a result, the number of ESD cases with a high risk of bleeding is also likely to increase. Therefore, ensuring the safety of ESD in patients at high risk of post‐ESD bleeding has become an urgent and unmet clinical need.

The technology of artificial intelligence (AI), particularly machine learning (ML) and deep learning, has advanced rapidly and is increasingly being applied to predict various clinical outcomes [16]. An AI‐based predictive model for post‐ESD bleeding using deep learning has been reported, achieving an area under the curve (AUC) of 0.71 [17]. This state‐of‐the‐art technology has significant potential to accurately predict post‐ESD bleeding and ultimately improve patient management. However, in deep learning models, the algorithm automatically determines the weight parameters of variables, making it difficult to calculate the contribution of each variable (i.e., feature importance) [18]. Therefore, this presents a challenge in understanding how the model generates its predictions. In clinical practice, identifying key predictive factors enhances model interpretability. Interpretable models are generally preferred, as they enable clinicians to understand the reasoning behind predictions and make more informed decisions.

Recent advancements in ML techniques have introduced methods to improve the interpretability of predictive models by quantifying the contribution of variables to model predictions while maintaining a balance between accuracy and transparency [19]. To our knowledge, no ML‐based predictive model for post‐ESD bleeding that considers variable contributions has yet been reported. Based on recent developments, we hypothesized that ML could facilitate the development of accurate and interpretable models, potentially outperforming traditional statistical models in predicting post‐ESD bleeding.

Therefore, this study aimed to develop ML predictive models for post‐ESD bleeding based on multicenter data, compare these with a non‐ML model and identify key factors associated with post‐ESD bleeding through feature importance analysis.

## 2 Method

### 2.1 Patients

This retrospective, multicenter cohort study was conducted at Niigata University Medical and Dental Hospital and Koseiren Murakami General Hospital. Patients who underwent ESD for early GC between 2012 and 2023 were included in this study (Figure S1). As in previous studies, patients with a history of gastric surgery were excluded due to altered hemodynamics and anatomy in the remnant stomach, which could complicate model development [20, 21]. Furthermore, cases involving the simultaneous resection of multiple lesions were excluded, as such cases also complicate the analysis. Consent for participation was obtained using an opt‐out process. The study protocol was approved by the institutional review boards of the participating institutions, coordinated through the central institutional review board at Niigata University (approval number: 2023–0252), and conducted following the principles of the Declaration of Helsinki.

### 2.2 ESD Procedure, Treatment Course, and Definition of Post‐ESD Bleeding

According to the Japanese gastric cancer treatment guideline, ESD was performed for lesions that met the criteria for absolute indication; in elderly patients or those with severe comorbidities, ESD was also performed for lesions meeting relative indication [22].

Antithrombotic agents, including DOACs (apixaban, edoxaban, dabigatran, and rivaroxaban), warfarin, and P2Y12RA, were discontinued prior to ESD following hospital regulations. Heparin bridging was used for patients with a high risk of thrombosis. For those on P2Y12RA, ESD was performed after either drug discontinuation, heparin bridging, or substitution with low‐dose aspirin, depending on the cases. Patients on low‐dose aspirin underwent ESD without discontinuation of the medication. The detailed protocols for the perioperative management of each antithrombotic agent are summarized in Figure S2.

Food intake was withheld on the day of ESD, and the procedure was performed as previously described [23, 24]. After GC resection, visible blood vessels in the ulcer base were coagulated using Coagrasper (Olympus, Tokyo, Japan), a commonly used hemostatic forceps. The ulcer was not closed and was left open.

An intravenous proton pump inhibitor (PPI)—lansoprazole 20 mg or omeprazole 20 mg, both administered twice daily—was administered for 2 days. On the third day, oral intake was resumed, and the PPI was switched to oral medication (vonoprazan 20 mg, esomeprazole 20 mg, lansoprazole 30 mg, or rabeprazole 20 mg). In a typical case, patients were discharged on the seventh day after ESD. Follow‐up endoscopy was performed to confirm ulcer scarring 3 months after ESD, and oral PPI treatment was continued until then.

Urgent endoscopy was performed in cases with suggestive findings of bleeding, indicated by signs such as hematemesis, melena, or decreased hemoglobin levels. ‘Overall post‐ESD bleeding’ was defined as bleeding confirmed to originate from the ESD ulcer base during emergency endoscopy, occurring within 28 days after ESD in accordance with definitions used in previous studies [20, 25].

Overall post‐ESD bleeding was further classified into two groups: “early bleeding” (occurring ≤ 7 days after ESD) and “late bleeding” (occurring > 7 days after ESD). This classification is based on the typical 7‐day hospital stay following ESD at our institutions. Late bleeding may occur after discharge, potentially resulting in treatment delays and worsening clinical outcomes. Therefore, the timing of post‐ESD bleeding should be carefully monitored.

### 2.3 Data Collection

Patient characteristics, including age, sex, comorbidities, and medications, were collected from medical records with reference to previous studies on post‐ESD bleeding [20, 26]. Patients were considered to have hypertension or diabetes mellitus if they were taking any medications for these conditions. The use of DOACs, warfarin, and antiplatelet agents (P2Y12RA, low‐dose aspirin, and cilostazol) was also investigated. In addition, the use of heparin bridging before the procedure was investigated in patients using antithrombotic agents. Endoscopic findings, such as the location of GC and tumor size, were investigated using endoscopic and histological databases at each hospital. A tumor size threshold of > 30 mm was adopted following a previous study and was subsequently used in the analyses [20]. Pathological diagnosis was conducted in accordance with the Japanese gastric cancer treatment guideline [22].

### 2.4 Construction of Predictive Models

The data set was randomly partitioned into two subsets: 70% for model development (training data set) and 30% for out‐of‐sample testing (test data set). ML predictive models were constructed using 20 pre‐ and peri‐treatment variables listed in Table S1 to identify patients at high risk of post‐ESD bleeding. The types of acid suppression therapy (PPIs or potassium‐competitive acid blocker [P‐CAB]) were not included in the models for the following reasons: (1) various drugs are used, and the route of administration (e.g., intravenous vs. oral) changes throughout the clinical course, complicating the modeling process, and (2) previous studies have reported that the type of PPI or P‐CAB is not associated with the incidence of post‐ESD bleeding [27, 28].

First, based on the training data set, a basic ML model was developed to predict the occurrence of overall post‐ESD bleeding. Python (version 3.13) was used for model development, and Categorical Boosting, a commonly used ML algorithm for categorical data, was employed [29]. Next, an advanced ML model was developed to differentiate between patients without post‐ESD bleeding, those with early bleeding, and those with late bleeding. Further details on the development of the ML models are provided in Supporting Information S1.

Shapley Additive explanations (SHAP) values were used to assess how each variable contributed to ML model predictions [30]. This enabled the identification of key factors associated with post‐ESD bleeding. SHAP interaction values were also calculated to evaluate how interactions between variables—such as combined medication use or the interplay between specific drugs and comorbidities—influenced the ML model's predictions [31].

To compare with the basic ML model, a non‐ML predictive model was developed using logistic regression analysis on the training data set. Each significant variable was assigned a score, and the total score was used as a risk score to predict post‐ESD bleeding. This score‐based prediction was defined as the non‐ML model. Details are provided in Supporting Information S1.

Model performance was evaluated by generating a receiver operating characteristic (ROC) curve and calculating the AUC using the test data set. Sensitivity, specificity, positive predictive value (PPV), and negative predictive value (NPV) were also calculated to evaluate each model. These metrics were compared between the basic ML and non‐ML models.

Furthermore, as the basic ML model provides outputs as continuous probabilities for overall post‐ESD bleeding, patients in the test data set were stratified into low (< 50%), intermediate (50%–80%), and high (≥ 80%) probability groups based on the predicted probabilities. The risks of overall post‐ESD bleeding were then evaluated across these groups.

Using the same process as the basic ML model, an advanced ML model was developed to differentiate between patients without post‐ESD bleeding, those with early bleeding, and those with late bleeding.

### 2.5 Statistical Analysis

Continuous variables were expressed as the median (interquartile range [IQR]), and categorical variables as the number (percentage). Univariate and multivariate logistic regression analyses were conducted to detect variables associated with overall post‐ESD bleeding. Variables with *p* < 0.05 in the univariate analysis were included in the multivariate analysis. The DeLong test compared the AUC between the non‐ML and basic ML models. McNemar's test was used to compare sensitivity and specificity, while the bootstrap method was employed for comparing PPV and NPV. The Wald test was applied to compare odds ratios (OR) among the probability groups. Statistical significance was set at *p* < 0.05. EZR (Saitama Medical Center, Jichi Medical University, Saitama, Japan) was used for statistical analysis [32].

## 3 Results

### 3.1 Baseline Characteristics of the Patients

A total of 1084 patients (mean age: 75 years [IQR: 70–79]; 815 males, 269 females) were included. Post‐ESD bleeding occurred in 63 patients (5.8%): 50 with early bleeding (4.6%) and 13 with late bleeding (1.2%) (Table 1 and Figure S1). The median time to bleeding was 6 days (IQR: 2–7).

**TABLE 1:** Baseline characteristics of 1084 patients who underwent ESD for early gastric cancer.

	Total (*N* = 1083)	Non‐bleeding (*n* = 1021)	Overall post‐ESD bleeding (*n* = 63)	*p* value
Age, median (IQR), years	75.0 (70.0–79.0)	75.0 (70.0–79.0)	77.0 (71.0–81.0)	0.04
Female sex, *n* (%)	269 (24.8)	255 (25.0)	14 (22.2)	0.62
Early bleeding, *n* (%)	50 (4.6)	n.a.	50 (79.4)	n.a.
Late bleeding, *n* (%)	13 (1.2)	n.a.	13 (20.6)	n.a.
Period from ESD to bleeding, median (IQR), days	6 (2–7)	n.a.	6 (2–7)	n.a.
Comorbidities				
Hypertension, *n* (%)	630 (58.1)	590 (59.8)	40 (65.6)	0.37
Diabetes mellitus, *n* (%)	192 (17.7)	180 (18.2)	12 (19.7)	0.78
Atrial fibrillation, *n* (%)	247 (22.8)	209 (21.2)	38 (62.3)	< 0.01
Valvular disease, *n* (%)	93 (8.6)	86 (8.7)	7 (11.5)	0.85
Ischemic heart disease, *n* (%)	78 (7.2)	72 (7.3)	6 (9.8)	0.46
Chronic heart failure, *n* (%)	244 (22.5)	229 (23.2)	15 (24.6)	0.80
Chronic renal failure on HD, *n* (%)	18 (1.7)	15 (1.5)	3 (4.9)	0.047
Liver cirrhosis, *n* (%)	24 (2.2)	23 (2.3)	1 (1.6)	0.73
DVT/PE, *n* (%)	39 (3.6)	37 (3.7)	2 (3.3)	0.46
Antithrombotic agents				
DOAC, *n* (%)	117 (10.8)	92 (9.0)	25 (39.7)	< 0.01
Low‐dose aspirin, *n* (%)	71 (6.5)	68 (6.7)	3 (4.8)	0.55
Cilostazol, *n* (%)	17 (1.6)	16 (1.6)	1 (1.6)	0.99
P2Y12RA, *n* (%)	37 (3.4)	29 (2.8)	8 (12.7)	< 0.01
Warfarin, *n* (%)	66 (6.1)	49 (4.8)	17 (27.0)	< 0.01
Heparin bridge, *n* (%)	130 (12.0)	105 (10.3)	25 (39.7)	< 0.01
Lesion and procedure				
Location (L/M/U)	491/432/161	460/409/152	31/23/9	0.81
Ulceration, *n* (%)	105 (9.7)	102 (10.0)	3 (4.8)	0.17
Undifferentiated type, *n* (%)	34 (3.1)	34 (3.3)	0 (0.0)	0.14
Tumor size > 30 mm, *n* (%)	92 (93.0)	88 (8.9)	4 (6.6)	0.53
En bloc resection, *n* (%)	1071 (98.8)	1010 (98.9)	61 (96.8)	0.14
Invasion depth, SM, *n* (%)	95 (8.8)	93 (9.1)	2 (3.2)	0.11
Lymphovascular involvement, *n* (%)	32 (3.0)	30 (3.0)	2 (3.2)	0.91

Abbreviations: DOAC, direct oral anticoagulant; DVT, deep venous thrombosis; ESD, endoscopic submucosal dissection; HD, hemodialysis; PE, pulmonary embolism; P2Y12 RA, P2Y12 receptor antagonists; SM, submucosa.

Patients with bleeding were significantly older than those without (77.0 vs. 75.0 years, *p* = 0.04). Comorbidities such as Af (62.3% vs. 21.2%, *p* < 0.01) and chronic renal failure on hemodialysis (4.9% vs. 1.5%, *p* = 0.047) were more common in the bleeding group. The use of antithrombotic agents was also more frequent in the bleeding group, including DOAC (39.7% vs. 10.3%), P2Y12RA (12.7% vs. 2.8%), warfarin (27.0% vs. 4.8%), and heparin bridging (39.7% vs. 10.3%) (all *p* < 0.01). There were no significant differences in endoscopic or histological findings between the groups.

### 3.2 Results of Predictive Models

**FIGURE 1:**
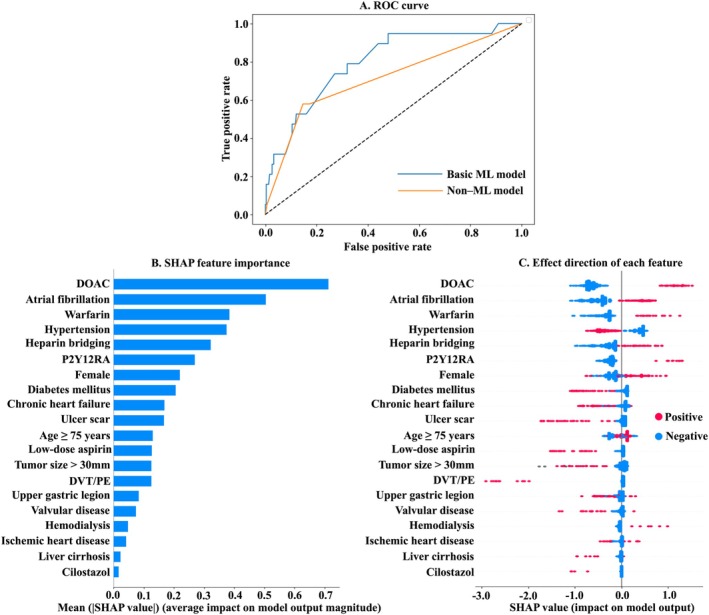
Prediction of overall post‐ESD bleeding –results of the basic ML model. (A) ROC curves for overall post‐ESD bleeding (B) SHAP importance values for overall post‐ESD bleeding prediction, presented as a bar graph illustrating the strength of influence of each variable on the prediction. (C) A beeswarm plot displaying the contribution of each variable to the prediction outcome. Red points indicate a positive prediction (presence of bleeding), while blue points indicate a negative prediction (absence of bleeding). The horizontal axis represents the SHAP value. AUC, area under the curve; DOAC, direct oral anticoagulant; DVT, deep venous thrombosis; ESD, endoscopic submucosal dissection; ML, machine learning; PE, pulmonary embolism; P2Y12RA, P2Y12 receptor antagonist; ROC, receiver‐operating characteristic; SHAP, Shapley Additive explanations.

The basic ML model predicting overall post‐ESD bleeding demonstrated good performance with an AUC of 0.80 (Table 2). In the non‐ML model, DOAC, P2Y12RA, and warfarin were assigned 6, 4, and 6 points, respectively (Table S1), yielding an AUC of 0.71; the basic ML model has significantly better AUC than that of the non‐ML model (*p* = 0.03) (Table 2 and Figure 1). The ML model showed a trend toward higher sensitivity (0.74 vs. 0.58, *p* = 0.58) and NPV (0.98 vs. 0.97, *p* = 0.59) compared with the non‐ML model, although the differences were not statistically significant. In contrast, the specificity of the basic ML model was significantly lower (0.68 vs. 0.85, *p* < 0.01), and PPV tended to be lower as well, although not statistically significant (0.12 vs. 0.20, *p* = 0.19). These findings suggest that the basic ML model is better suited for screening purposes, while the non‐ML model is more appropriate for confirmatory diagnosis.

**TABLE 2:** Comparison of predictive performance between the basic ML model and non–ML model.

	Basic ML model	Non–ML model	*p* value*
AUC (95% CI)	0.80 (0.68–0.89)	0.71 (0.58–0.82)	0.03
Sensitivity (95% CI)	0.74 (0.53–0.91)	0.58 (0.33–0.79)	0.58
Specificity (95% CI)	0.68 (0.62–0.73)	0.85 (0.81–0.89)	< 0.01
PPV (95% CI)	0.12 (0.07–0.19)	0.20 (0.09–0.31)	0.19
NPV (95% CI)	0.98 (0.95–1.00)	0.97 (0.95–0.99)	0.59

Abbreviations: AUC, area under the curve; ML, machine learning; NPV, negative predictive value; PPV, positive predictive value.

* The Delong test was used for the AUC comparison. MacNemar's test was used to compare sensitivity and specificity, while the bootstrap mehod was employed for comparing PPV and NPV.

**FIGURE 2:**
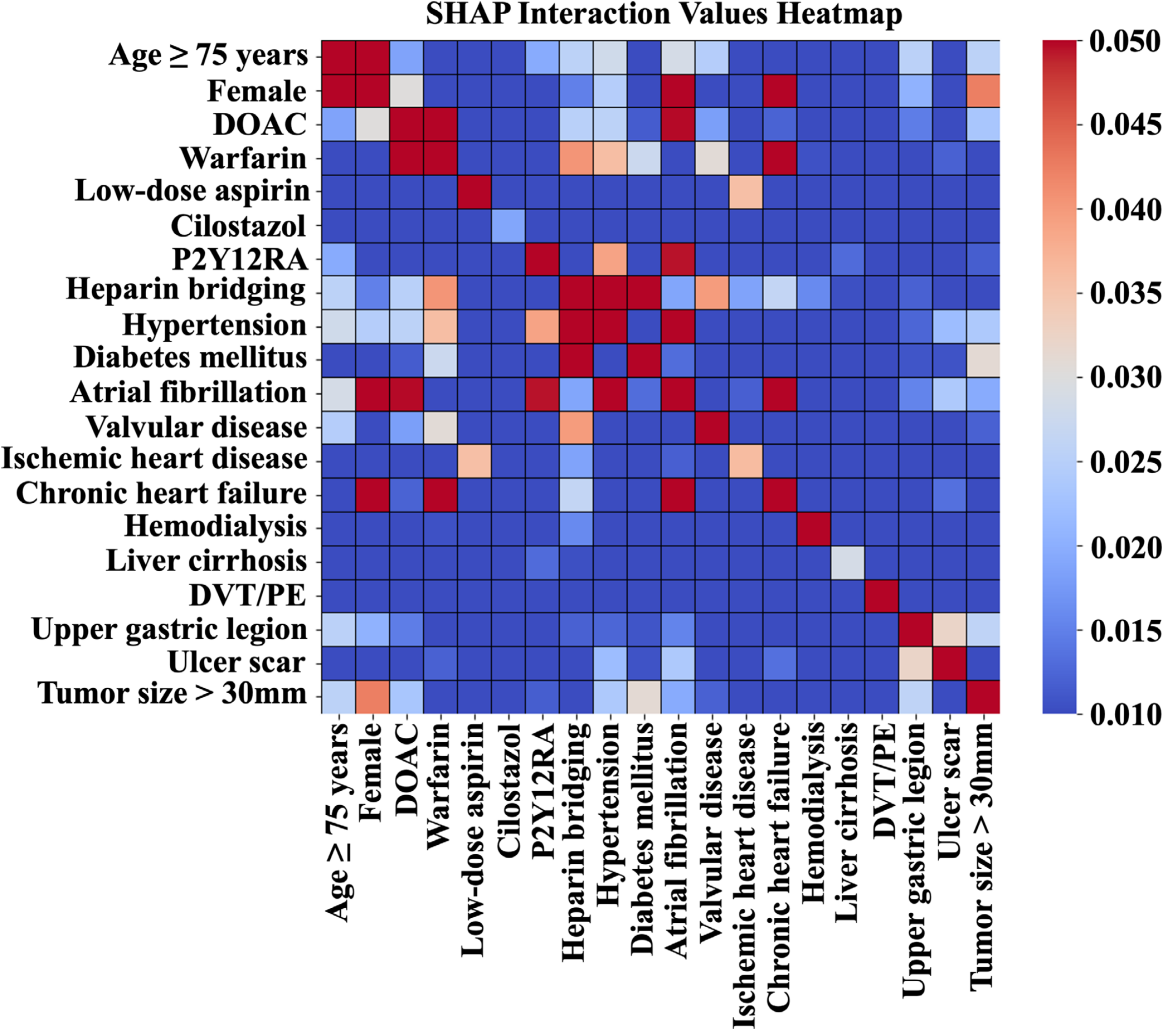
Interaction of each variable in the prediction of total bleeding. The heatmap revealed the interactions between variables, with the color bar representing the SHAP interaction values. These values were derived from the SHAP values of individual variables, which measure the impact of each variable on the model output. DOAC, direct oral anticoagulant; DVT, deep venous thrombosis; PE, pulmonary embolism; P2Y12RA, P2Y12 receptor antagonist; SHAP, Shapley Additive explanations.

The SHAP feature importance values identified the use of DOACs as the most influential factor in predicting overall post‐ESD bleeding (Figure 1B,C). Af and warfarin were also associated with an increased risk of bleeding, while hypertension was an important predictor associated with a lower likelihood of bleeding. Among the top‐ranked variables in the SHAP feature importance analysis, the SHAP interaction analysis demonstrated that interactions between Af and DOAC use contributed to the model's prediction of post‐ESD bleeding (Figure 2). Furthermore, Af exhibited interactions with hypertension and P2Y12RA use, which collectively exerted a strong influence on the model's prediction of post‐ESD bleeding.

When stratified by the bleeding probability estimated by the basic ML model, the bleeding rates were 2.3%, 8.8%, and 28.6% in the low‐, intermediate‐, and high‐probability groups, respectively (Table 3). Compared to the low‐probability group, the odds of post‐ESD bleeding were significantly higher in the intermediate‐probability group (OR 4.03, *p* = 0.02) and the high‐probability group (OR 16.7, *p* < 0.01), suggesting that the ML‐predicted bleeding probability serves as a useful marker for stratifying post‐ESD bleeding risk.

**TABLE 3:** Bleeding rate based on the bleeding probability calculated by the basic ML model.

Probability category	Bleeding probability	Bleeding rate, % (n/N)	OR (95% CI)	p value
Low	< 50%	2.3 (5/214)	Ref	
Intermediate	50%–80%	8.8 (8/91)	4.03 (1.28–12.7)	0.02
High	≥ 80	28.6 (6/21)	16.7 (4.57–61.2)	< 0.01

Abbreviations: CI, confidence interval; OR, odds ratio.

**FIGURE 3:**
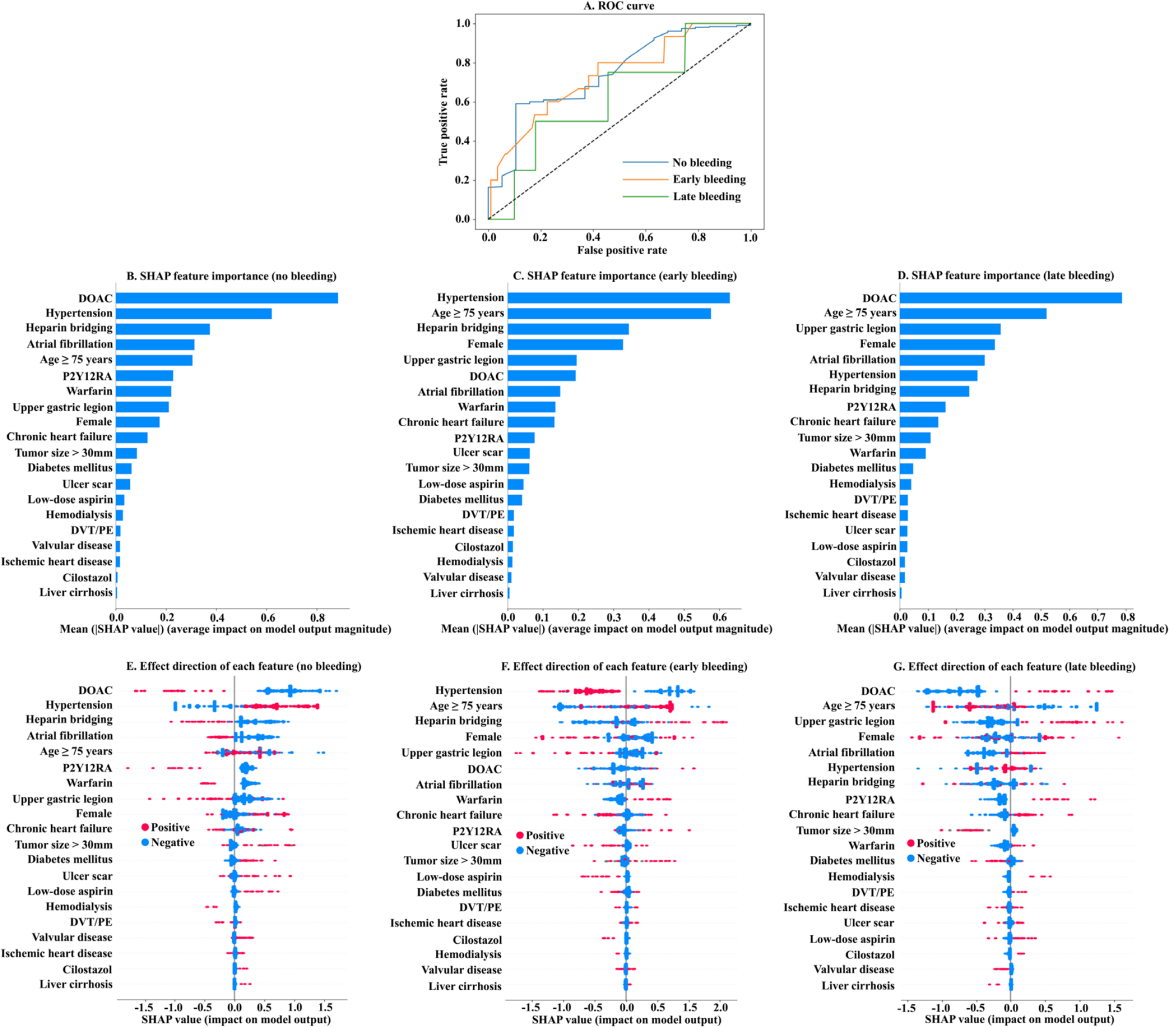
Prediction of early and late bleeding –results of the advanced ML model. (A) ROC curve for non‐bleeding, early bleeding, and late bleeding. (B, C, D) SHAP importance values for non‐bleeding, early bleeding, and late bleeding. (E, F, G) Beeswarm plots displaying the contribution of each variable to the prediction outcome. The horizontal axis represents the SHAP value. AUC, area under the curve; DOAC, direct oral anticoagulant; DVT, deep venous thrombosis; ESD, endoscopic submucosal dissection; ML, machine learning; PE, pulmonary embolism; P2Y12RA, P2Y12 receptor antagonist; ROC, receiver‐operating characteristic; SHAP, Shapley Additive explanation.

In contrast, the performance of the advanced ML model, which differentiated between non‐bleeding, early bleeding, and late bleeding, was suboptimal. The AUC values for identifying non‐bleeding, early bleeding, and late bleeding were 0.75, 0.74, and 0.63, respectively (Figure 3A and Table 4). SHAP feature importance analysis indicated that DOAC use and hypertension were among the top contributing factors across all three outcomes (Figure 3B–G). However, the importance of these variables should be interpreted with caution due to the limited predictive performance of the advanced ML model. SHAP interaction results are summarized in Figure S3.

**TABLE 4:** Results of machine‐learning predictive models for bleeding after endoscopic submucosal dissection.

	Prediction for non‐bleeding	Prediction for early bleeding	Prediction for late bleeding
AUC (95% CI)	0.75 (0.63–0.85)	0.74 (0.60–0.86)	0.63 (0.26–0.89)
Sensitivity (95% CI)	0.70 (0.65–0.75)	0.53 (0.28–0.79)	0.00 (0.00–0.00)
Specificity (95% CI)	0.58 (0.36–0.80)	0.79 (0.74–0.83)	0.91 (0.88–0.94)
PPV (95% CI)	0.96 (0.94–0.99)	0.11 (0.04–0.18)	0.00 (0.00–0.00)
NPV (95% CI)	0.11 (0.05–0.17)	0.97 (0.95–0.99)	0.99 (0.97–1.00)

*Note:* The performance of the advanced ML model was evaluated using one‐vs‐all analysis, a strategy commonly applied in multi‐class classification tasks. This approach transforms the multi‐class problem into multiple binary classification problems, where each class is treated as the ‘positive’ class, and all other classes are combined into a single ‘negative’ class.

Abbreviations: AUC, area under the curve; ML, machine learning; NPV, negative predictive value; PPV, positive predictive value.

## 4 Discussion

Our basic ML model successfully predicted overall post‐ESD bleeding, with antithrombotic agents, Af, and hypertension identified as key predictors. The AUC of the basic ML model was significantly higher than that of the non‐ML model. Moreover, the ML model's ability to estimate individualized bleeding probabilities enabled meaningful risk stratification. The model also uncovered important interactions among key variables that contributed to its predictive performance. However, the performance of the advanced ML model was suboptimal, and developing a more sophisticated model capable of accurately predicting the timing of bleeding remains a significant challenge.

Regarding the prediction of post‐ESD bleeding, a Japanese group developed a non‐AI predictive model based on rigorous statistical methodology [20, 33]. The AUC values for this model, as well as for deep learning models, are approximately 0.70 [17, 20]. Although differences in patient cohorts make direct comparisons challenging, our ML model achieved a high AUC of 0.80, which was significantly higher than that of our non‐ML model. The sensitivity of our ML model also tended to be higher than that of the non‐ML model. This is particularly important because, in predicting post‐ESD bleeding, minimizing missed cases is critical due to the potentially serious consequences of bleeding. Therefore, we consider the ML‐based model more suitable for screening purposes. A notable advantage of the ML model is its ability to provide individualized bleeding probabilities, enabling effective risk stratification. When our model predicts a high probability of bleeding, proactive interventions such as prophylactic ulcer closure may help reduce the risk of post‐ESD bleeding [34]. Although the PPV of our ML model is relatively low, which may lead to some false positives and result in unnecessary prophylactic interventions for patients who would not have experienced bleeding, such interventions are minimally invasive. Considering the potential burden on patients in the event of actual bleeding, we consider that a certain degree of over‐triage is acceptable.

The strength of our ML model lies in its interpretability, as demonstrated by SHAP feature importance values. The previous non‐AI model relied on factors such as the use of antithrombotic agents, a well‐established risk factor for post‐ESD bleeding [20]. For instance, the risk of bleeding increases to 8.7%–20.8% in patients taking DOACs and to 8.3% in those taking P2Y12RA [35, 36]. Consistent with these findings, our ML model also identified antithrombotic agents as key predictors, supporting its clinical relevance. Af also emerged as a key factor, likely reflecting confounding from antithrombotic use. While confounding is often a concern in statistical analysis, ML algorithms automatically adjust variable weights, allowing confounding variables to be incorporated into the model. This helps mitigate confounding effects and enhances predictive accuracy [37].

Interestingly, hypertension was associated with a lower bleeding risk in the ML analysis, which is contrary to a previous finding [11]. In this study, patients were considered to have hypertension if they were taking antihypertensive medications, meaning that all patients with hypertension were receiving treatment. Therefore, it is possible that their intraoperative and postoperative blood pressure remained paradoxically more stable than those without hypertension, potentially contributing to the reduced bleeding risk. To determine whether blood pressure truly has no effect on bleeding, it would be necessary to monitor intraoperative and postoperative blood pressure fluctuations. However, because this was a retrospective study, intraoperative and postoperative blood pressure data were not systematically collected, limiting our ability to fully assess the influence of blood pressure stability on bleeding risk.

Regarding interactions between variables, Af was found to interact with key bleeding‐related medications such as DOAC and P2Y12RA, as well as with chronic heart failure. These variables are likely confounded, and the SHAP interaction analysis likely reflects their combined effects on the prediction after accounting for such confounding.

The advanced ML model did not achieve sufficient predictive accuracy, likely due to the small sample size relative to its complexity, particularly the limited number of late bleeding cases. A key strength of ML lies in its flexibility—new variables can be easily incorporated, and the model can continue to learn and improve over time. Given the clinical importance of the timing of bleeding after ESD, it is warranted that the sample size be increased and a more robust model capable of distinguishing bleeding timing be developed.

Our study has several limitations. First, due to the retrospective nature of this study, certain information, such as *Helicobacter pylori* infection status, the severity of chronic gastritis, and intraoperative or postoperative blood pressure, was unavailable. Incorporating such variables into the ML model may help build a more accurate predictive model. Second, while we evaluated the performance of our ML models using standard internal validation methods, external validation with an independent or prospective cohort is essential before clinical implementation. Such validation would ensure the generalizability and robustness of the model across diverse patient populations and clinical settings, which is critical for its safe and effective use in practice. Finally, the relatively low incidence of post‐ESD bleeding, particularly late bleeding, may have led to imbalanced data, potentially affecting model training and performance.

In conclusion, the ML predictive model could successfully predict the occurrence of post‐ESD bleeding and stratify bleeding risks. By analyzing feature importance, we identified the factors the model prioritized in making predictions, thus making it interpretable. With further validation, our predictive model has the potential to guide prophylactic measures to reduce the risk of bleeding after ESD.

## Conflicts of Interest

The authors declare no conflicts of interest.

## Supporting information


**FIGURE S1.** **Study population** This study included 1084 patients who underwent endoscopic submucosal dissection (ESD) for early gastric cancer. Of these, 63 patients experienced post‐ESD bleeding: 50 experienced early bleeding (≤ 7 days after ESD), and 13 experienced late bleeding (> 7 days after ESD).

**FIGURE S2.** **Protocols for the perioperative management of antithrombotic agents** The figure illustrated the detailed protocol for managing antithrombotic agents during the perioperative period and summarized the guidelines for food intake and PPI administration. DOAC, direct oral anticoagulant; ESD, endoscopic submucosal dissection; PPI, proton pump inhibitor.

**FIGURE S3.** **Interactions of each variable in the prediction of non‐, early, and late bleeding** The heatmap revealed the interactions between variables, with the color bar representing the SHAP interaction values. These values were derived from the SHAP values of the individual variables, which measured the impact of each variable on the model’s output. DOAC, direct oral anticoagulant; DVT, deep venous thrombosis; PE, pulmonary embolism; P2Y12RA, P2Y12 receptor antagonist; SHAP, Shapley Additive explanations.

**Data S1.** Supporting Information.

**TABLE S1.** Logistic regression analysis of factors predicting post‐ESD bleeding in the training data set.

## Data Availability

The data that support the findings of this study are available on request from the corresponding author. The data are not publicly available due to privacy or ethical restrictions.

## References

[1] W.‐J. Yang , H.‐P. Zhao , Y. Yu , et al., “Updates on Global Epidemiology, Risk and Prognostic Factors of Gastric Cancer,” World Journal of Gastroenterology 29 (2023): 2452–2468. 37179585 10.3748/wjg.v29.i16.2452 PMC10167900

[2] S. Shichijo , N. Uedo , T. Kanesaka , et al., “Long‐Term Outcomes After Endoscopic Submucosal Dissection for Differentiated‐Type Early Gastric Cancer That Fulfilled Expanded Indication Criteria: A Prospective Cohort Study,” Journal of Gastroenterology and Hepatology 36 (2021): 664–670. 32663347 10.1111/jgh.15182 PMC7983953

[3] T. Gotoda and H.‐Y. Jung , “Endoscopic Resection (Endoscopic Mucosal Resection/Endoscopic Submucosal Dissection) for Early Gastric Cancer,” Digestive Endoscopy 25, no. Suppl 1 (2013): 55–63. 23362925 10.1111/den.12003

[4] T. Akasaka , T. Nishida , S. Tsutsui , et al., “Short‐Term Outcomes of Endoscopic Submucosal Dissection (ESD) for Early Gastric Neoplasm: Multicenter Survey by Osaka University ESD Study Group,” Digestive Endoscopy 23 (2011): 73–77. 21198921 10.1111/j.1443-1661.2010.01062.x

[5] M. Kato , T. Nishida , K. Yamamoto , et al., “Scheduled Endoscopic Surveillance Controls Secondary Cancer After Curative Endoscopic Resection for Early Gastric Cancer: A Multicentre Retrospective Cohort Study by Osaka University ESD Study Group,” Gut 62 (2013): 1425–1432. 22914298 10.1136/gutjnl-2011-301647

[6] H. Suzuki , I. Oda , S. Abe , et al., “High Rate of 5‐Year Survival Among Patients With Early Gastric Cancer Undergoing Curative Endoscopic Submucosal Dissection,” Gastric Cancer 19 (2016): 198–205. 25616808 10.1007/s10120-015-0469-0

[7] S. Mukai , S. Cho , T. Kotachi , et al., “Analysis of Delayed Bleeding After Endoscopic Submucosal Dissection for Gastric Epithelial Neoplasms,” Gastroenterology Research and Practice 2012 (2012): 875323. 22536221 10.1155/2012/875323 PMC3296301

[8] H. Tomida , T. Yoshio , K. Igarashi , et al., “Influence of Anticoagulants on the Risk of Delayed Bleeding After Gastric Endoscopic Submucosal Dissection: A Multicenter Retrospective Study,” Gastric Cancer 24 (2021): 179–189. 32683602 10.1007/s10120-020-01105-0

[9] R. Koh , K. Hirasawa , S. Yahara , et al., “Antithrombotic Drugs Are Risk Factors for Delayed Postoperative Bleeding After Endoscopic Submucosal Dissection for Gastric Neoplasms,” Gastrointestinal Endoscopy 78 (2013): 476–483. 23622974 10.1016/j.gie.2013.03.008

[10] T. Takeuchi , K. Ota , S. Harada , et al., “The Postoperative Bleeding Rate and Its Risk Factors in Patients on Antithrombotic Therapy Who Undergo Gastric Endoscopic Submucosal Dissection,” BMC Gastroenterology 13 (2013): 136. 24010587 10.1186/1471-230X-13-136 PMC3844538

[11] M. Ebi , T. Shimura , H. Nishiwaki , et al., “Management of Systolic Blood Pressure After Endoscopic Submucosal Dissection Is Crucial for Prevention of Post‐ESD Gastric Bleeding,” European Journal of Gastroenterology & Hepatology 26 (2014): 504–509. 24589830 10.1097/MEG.0000000000000072

[12] Y. Liu , S. Kobayashi , K. Karako , P. Song , and W. Tang , “The Latest Policies, Practices, and Hotspots in Research in Conjunction With the Aging of Japan's Population,” Bioscience Trends 18 (2024): 219–223. 38866487 10.5582/bst.2024.01150

[13] N. Tsuji , Y. Takahashi , M. Sakai , et al., “Trend of Anticoagulant Therapy in Elderly Patients With Atrial Fibrillation Considering Risks of Cerebral Infarction and Bleeding,” Scientific Reports 13 (2023): 192. 36604482 10.1038/s41598-022-26741-7 PMC9814101

[14] M. G. Lansberg , M. J. O'Donnell , P. Khatri , et al., “Antithrombotic and Thrombolytic Therapy for Ischemic Stroke: Antithrombotic Therapy and Prevention of Thrombosis, 9th Ed: American College of Chest Physicians Evidence‐Based Clinical Practice Guidelines,” Chest 141 (2012): e601S–e636S. 22315273 10.1378/chest.11-2302 PMC3278065

[15] G. N. Levine , E. R. Bates , J. C. Blankenship , et al., “2011 ACCF/AHA/SCAI Guideline for Percutaneous Coronary Intervention: A Report of the American College of Cardiology Foundation/American Heart Association Task Force on Practice Guidelines and the Society for Cardiovascular Angiography and Interventions,” Catheterization and Cardiovascular Interventions 82 (2013): E266–E355. 22065485 10.1002/ccd.23390

[16] K. Takahashi , H. Sato , Y. Shimamura , et al., “Achalasia Phenotypes and Prediction of Per‐Oral Endoscopic Myotomy Outcomes Using Machine Learning,” Digestive Endoscopy 36, no. 7 (2024): 789–800, 10.1111/den.14714. 37886891

[17] J. E. Na , Y. C. Lee , T. J. Kim , et al., “Utility of a Deep Learning Model and a Clinical Model for Predicting Bleeding After Endoscopic Submucosal Dissection in Patients With Early Gastric Cancer,” World Journal of Gastroenterology 28 (2022): 2721–2732. 35979158 10.3748/wjg.v28.i24.2721 PMC9260866

[18] R. Guidotti , A. Monreale , S. Ruggieri , F. Turini , F. Giannotti , and D. Pedreschi , “A Survey of Methods for Explaining Black Box Models,” ACM Computing Surveys 51 (2019): 1–42.

[19] G. Stiglic , P. Kocbek , N. Fijacko , M. Zitnik , K. Verbert , and L. Cilar , “Interpretability of Machine Learning‐Based Prediction Models in Healthcare,” WIREs Data Mining and Knowledge Discovery 10 (2020): e1379.

[20] W. Hatta , Y. Tsuji , T. Yoshio , et al., “Prediction Model of Bleeding After Endoscopic Submucosal Dissection for Early Gastric Cancer: BEST‐J Score,” Gut 70 (2021): 476–484. 32499390 10.1136/gutjnl-2019-319926 PMC7873424

[21] Y. Kagawa , M. Fukuzawa , M. Sugimoto , et al., “Validation of the BEST‐J Score, a Prediction Model for Bleeding After Endoscopic Submucosal Dissection for Early Gastric Cancer: A Multicenter Retrospective Observational Study,” Surgical Endoscopy 36 (2022): 7240–7249. 35194665 10.1007/s00464-022-09096-y

[22] Japanese Gastric Cancer Association , “Japanese Gastric Cancer Treatment Guidelines 2021 (6th Edition),” Gastric Cancer 26 (2023): 1–25. 36342574 10.1007/s10120-022-01331-8 PMC9813208

[23] K. Okada , Y. Yamamoto , A. Kasuga , et al., “Risk Factors for Delayed Bleeding After Endoscopic Submucosal Dissection for Gastric Neoplasm,” Surgical Endoscopy 25 (2011): 98–107. 20549245 10.1007/s00464-010-1137-4

[24] T. Yoshio , T. Nishida , N. Kawai , et al., “Gastric ESD Under Heparin Replacement at High‐Risk Patients of Thromboembolism Is Technically Feasible but has a High Risk of Delayed Bleeding: Osaka University ESD Study Group,” Gastroenterology Research and Practice 2013 (2013): 365830. 23843783 10.1155/2013/365830 PMC3697307

[25] S. Mochizuki , N. Uedo , I. Oda , et al., “Scheduled Second‐Look Endoscopy Is Not Recommended After Endoscopic Submucosal Dissection for Gastric Neoplasms (The SAFE Trial): A Multicentre Prospective Randomised Controlled Non‐Inferiority Trial,” Gut 64 (2015): 397–405. 25301853 10.1136/gutjnl-2014-307552

[26] T. Yoshio , H. Tomida , R. Iwasaki , et al., “Effect of Direct Oral Anticoagulants on the Risk of Delayed Bleeding After Gastric Endoscopic Submucosal Dissection,” Digestive Endoscopy 29 (2017): 686–694. 28295638 10.1111/den.12859

[27] H. Shibata , T. Fujiyoshi , N. Kawata , et al., “Vonoprazan or Proton Pump Inhibitor for Gastric Endoscopic Submucosal Dissection in Patients Taking Antithrombotic Agents,” Journal of Gastroenterology and Hepatology [Internet], ahead of print, May 27, (2025), 10.1111/jgh.17027. PMC12315787 40420803

[28] T. Uchiyama , T. Higurashi , H. Kuriyama , Y. Kondo , Y. Hata , and A. Nakajima , “Oral Esomeprazole vs Injectable Omeprazole for the Prevention of Hemorrhage After Endoscopic Submucosal Dissection,” World J Gastrointest Endosc 9 (2017): 514–520. 29085562 10.4253/wjge.v9.i10.514 PMC5648994

[29] L. Prokhorenkova , G. Gusev , A. Vorobev , A. V. Dorogush , and A. Gulin , “CatBoost: Unbiased Boosting With Categorical Features [Internet],” arXiv [cs.LG] (2017), http://arxiv.org/abs/1706.09516.

[30] S. M. Lundberg and S.‐I. Lee , “A Unified Approach to Interpreting Model Predictions,” in Proceedings of the 31st International Conference on Neural Information Processing Systems (Curran Associates Inc, 2017), 4765–4774.

[31] S. M. Lundberg , G. G. Erion , and S.‐I. Lee , “Consistent Individualized Feature Attribution for Tree Ensembles [Internet]. arXiv [cs.LG],” 2018, http://arxiv.org/abs/1802.03888.

[32] Y. Kanda , “Investigation of the Freely Available Easy‐To‐Use Software “EZR” for Medical Statistics,” Bone Marrow Transplantation 48 (2013): 452–458. 23208313 10.1038/bmt.2012.244 PMC3590441

[33] T. Okada , T. Mikamo , W. Hamamoto , et al., “Modified BEST‐J Score Model Predicts Bleeding After Endoscopic Submucosal Dissection With Fewer Factors,” Cancers (Basel) 14 (2022): 5555. 36428648 10.3390/cancers14225555 PMC9688376

[34] N. Kobayashi , H. Kobara , N. Nishiyama , et al., “Comparison of Endoscopic Closure Versus Non‐Closure for Post‐Gastric Endoscopic Submucosal Dissection Artificial Floor in Antithrombotic Therapy: A Propensity Score‐Matched Analysis,” Annals of Gastroenterology 36 (2023): 178–184. 36864933 10.20524/aog.2023.0771 PMC9932868

[35] R. Hirai , S. Kawano , S. Inoo , et al., “Postoperative Bleeding Risk After Gastric Endoscopic Submucosal Dissection in Patients Receiving a P2Y12 Receptor Antagonist,” Gut Liver 17 (2023): 404–411. 36172713 10.5009/gnl220196 PMC10191786

[36] M. Sugimoto , M. Murata , and T. Kawai , “Assessment of Delayed Bleeding After Endoscopic Submucosal Dissection of Early‐Stage Gastrointestinal Tumors in Patients Receiving Direct Oral Anticoagulants,” World Journal of Gastroenterology 29 (2023): 2916–2931. 37274799 10.3748/wjg.v29.i19.2916 PMC10237096

[37] P. Liu , X.‐J. Li , T. Zhang , and Y.‐H. Huang , “Comparison Between XGboost Model and Logistic Regression Model for Predicting Sepsis After Extremely Severe Burns,” Journal of International Medical Research 52 (2024): 3000605241247696. 38698505 10.1177/03000605241247696 PMC11067675

