# Supplementary material for: Machine‐Learning Prediction of Bleeding After Endoscopic Submucosal Dissection for Early Gastric Cancer: A Multicenter Study
Source: JGH Open. 2025 Jun 29;9(7):e70203. doi: 10.1002/jgh3.70203 (PMC12206847; doi:10.1002/jgh3.70203)
Supplement: Supplementary file 1 — FIGURE S1. Study population This study included 1084 patients who underwent endoscopic submucosal dissection (ESD) for early gastric cancer. Of these, 63 patients experienced post‐ESD bleeding: 50 experienced early bleeding (≤ 7 days after ESD), and 13 experienced late bleeding (> 7 days after ESD). [file JGH3-9-e70203-s001.pdf]

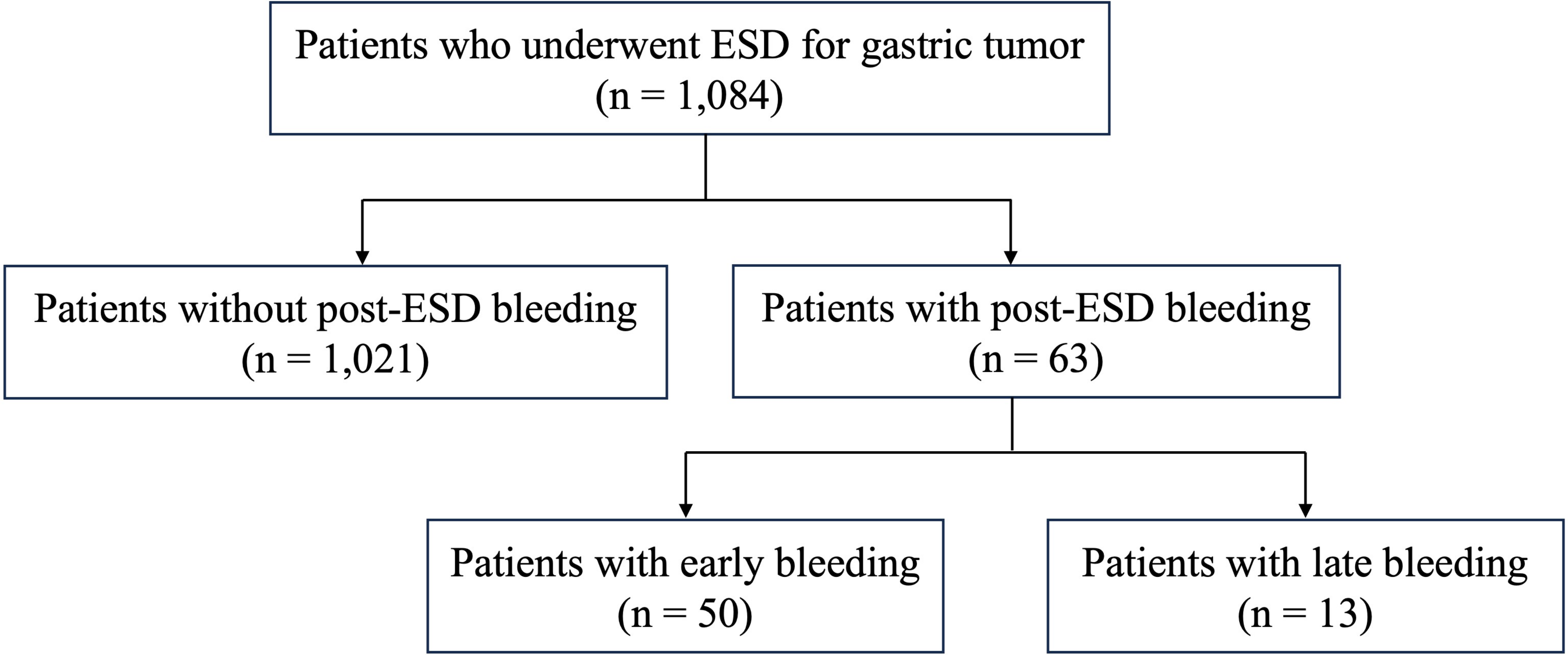
Patients who underwent ESD for gastric tumor
(n = 1,084)
Patients without post-ESD bleeding
(n = 1,021)
Patients with post-ESD bleeding
(n = 63)
Patients with early bleeding
(n = 50)
Patients with late bleeding
(n = 13)
