# Supplementary material for: Machine‐Learning Prediction of Bleeding After Endoscopic Submucosal Dissection for Early Gastric Cancer: A Multicenter Study
Source: JGH Open. 2025 Jun 29;9(7):e70203. doi: 10.1002/jgh3.70203 (PMC12206847; doi:10.1002/jgh3.70203)
Supplement: Supplementary file 2 — FIGURE S2. Protocols for the perioperative management of antithrombotic agents The figure illustrated the detailed protocol for managing antithrombotic agents during the perioperative period and summarized the guidelines for food intake and PPI administration. DOAC, direct oral anticoagulant; ESD, endoscopic submucosal dissection; PPI, proton pump inhibitor. [file JGH3-9-e70203-s005.pdf]

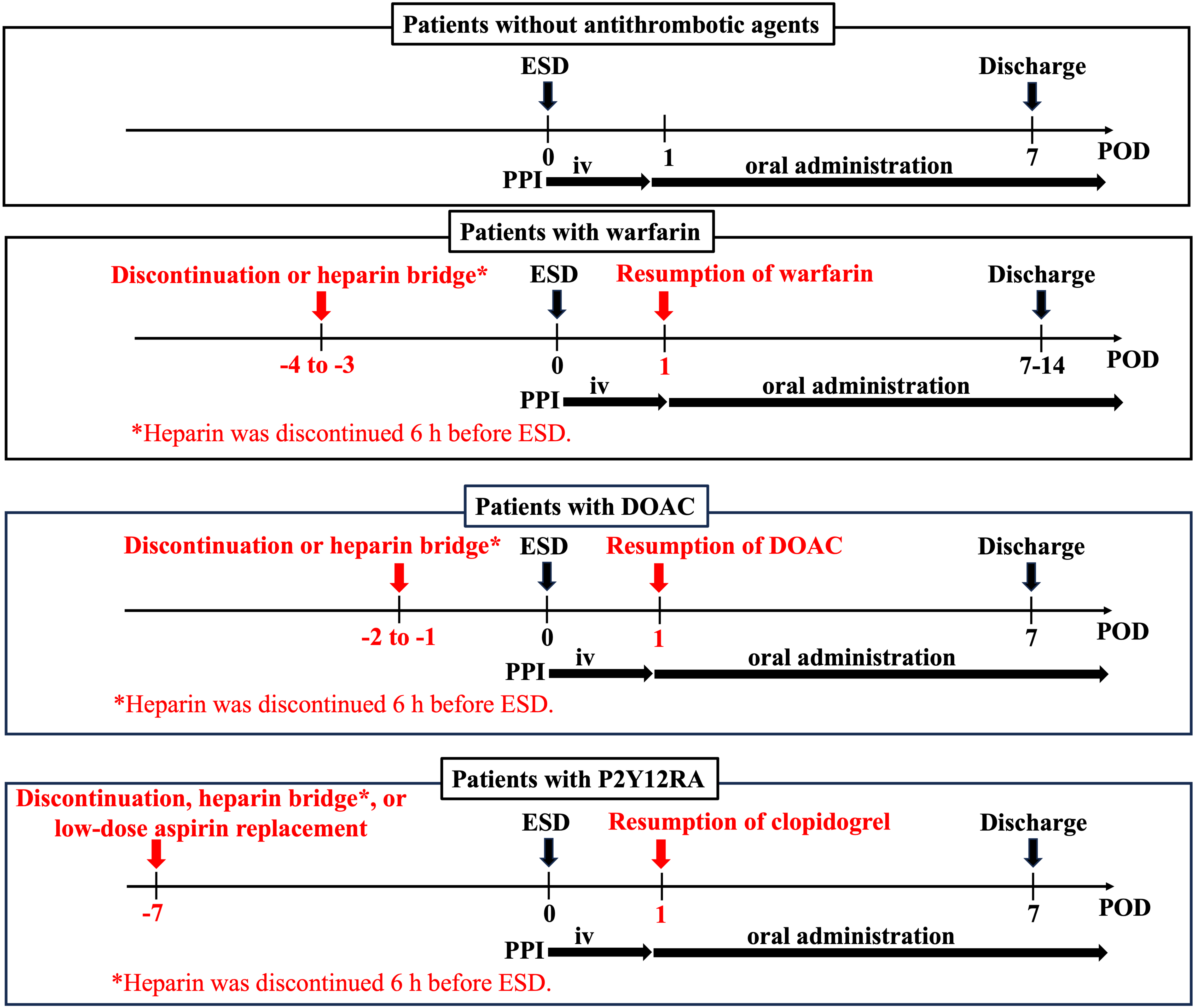
Patients without antithrombotic agents
ESD
Discharge
0
1
7
POD
PPI
iv
oral administration
Patients with warfarin
Discontinuation or heparin bridge*
ESD
Resumption of warfarin
Discharge
-4 to -3
0
1
7-14
POD
PPI
iv
oral administration
*Heparin was discontinued 6 h before ESD.
Patients with DOAC
Discontinuation or heparin bridge*
ESD
Resumption of DOAC
Discharge
-2 to -1
0
1
7
POD
PPI
iv
oral administration
*Heparin was discontinued 6 h before ESD.
Patients with P2Y12RA
Discontinuation, heparin bridge*, or
low-dose aspirin replacement
ESD
Resumption of clopidogrel
Discharge
-7
0
1
7
POD
PPI
iv
oral administration
*Heparin was discontinued 6 h before ESD.
