# Supplementary figures and images for: Machine‐Learning Prediction of Bleeding After Endoscopic Submucosal Dissection for Early Gastric Cancer: A Multicenter Study
Source: JGH Open. 2025 Jun 29;9(7):e70203. doi: 10.1002/jgh3.70203 (PMC12206847; doi:10.1002/jgh3.70203)

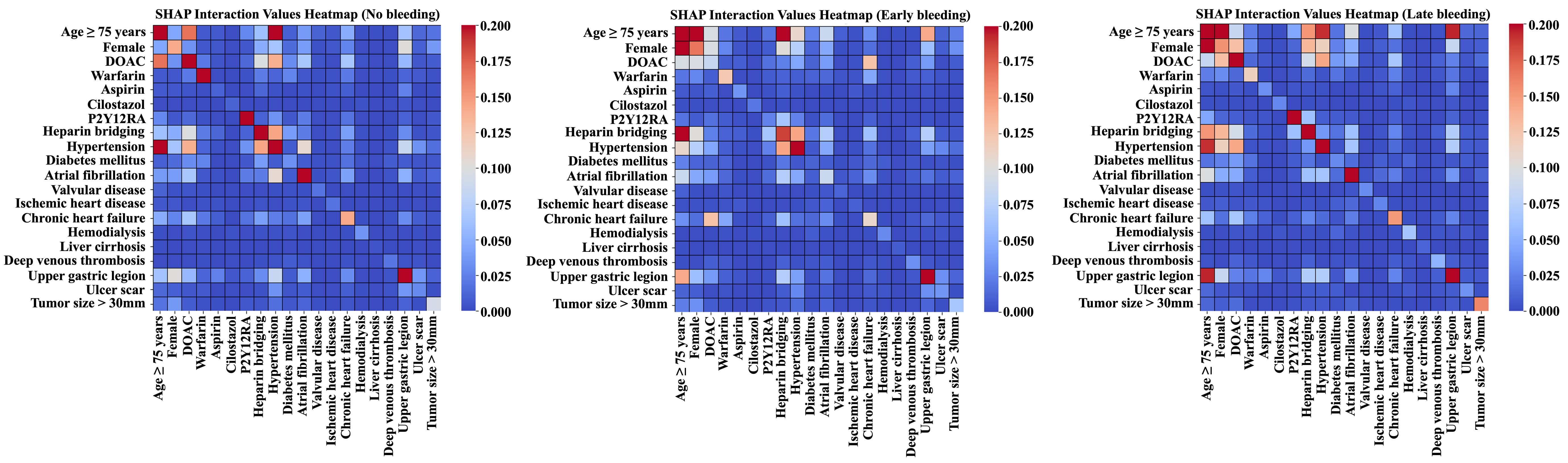

Supplement: Supplementary file 3 — FIGURE S3. Interactions of each variable in the prediction of non‐, early, and late bleeding The heatmap revealed the interactions between variables, with the color bar representing the SHAP interaction values. These values were derived from the SHAP values of the individual variables, which measured the impact of each variable on the model’s output. DOAC, direct oral anticoagulant; DVT, deep venous thrombosis; PE, pulmonary embolism; P2Y12RA, P2Y12 receptor antagonist; SHAP, Shapley Additive explanations. [file JGH3-9-e70203-s002.pdf]
