# Supplementary material for: Machine‐Learning Prediction of Bleeding After Endoscopic Submucosal Dissection for Early Gastric Cancer: A Multicenter Study
Source: JGH Open. 2025 Jun 29;9(7):e70203. doi: 10.1002/jgh3.70203 (PMC12206847; doi:10.1002/jgh3.70203)
Supplement: Supplementary file 4 — Data S1. Supporting Information. [file JGH3-9-e70203-s004.docx]

**Supporting Information S1**

**Detailed method of developing machine learning (ML) models**

The dataset was randomly partitioned into two subsets: 70% for model development (training dataset) and 30% for out-of-sample testing (test dataset), as shown in the below figure.

First, a basic ML model was developed to predict the occurrence of overall post-ESD bleeding using the training dataset. Eighteen pre- and peri-treatment variables listed in Table S1 were used for model construction. Python (version 3.13) was used for model development, and Categorical Boosting, a commonly used ML algorithm for categorical data, was employed [E1]. Grid Search with 10-fold cross-validation was applied to optimise the hyperparameters of the models, adjusting the learning rate and model complexity using the training dataset.

Using the same process as the basic ML model, an advanced ML model was also developed to differentiate between patients without post-ESD bleeding, those with early bleeding, and those with late bleeding.


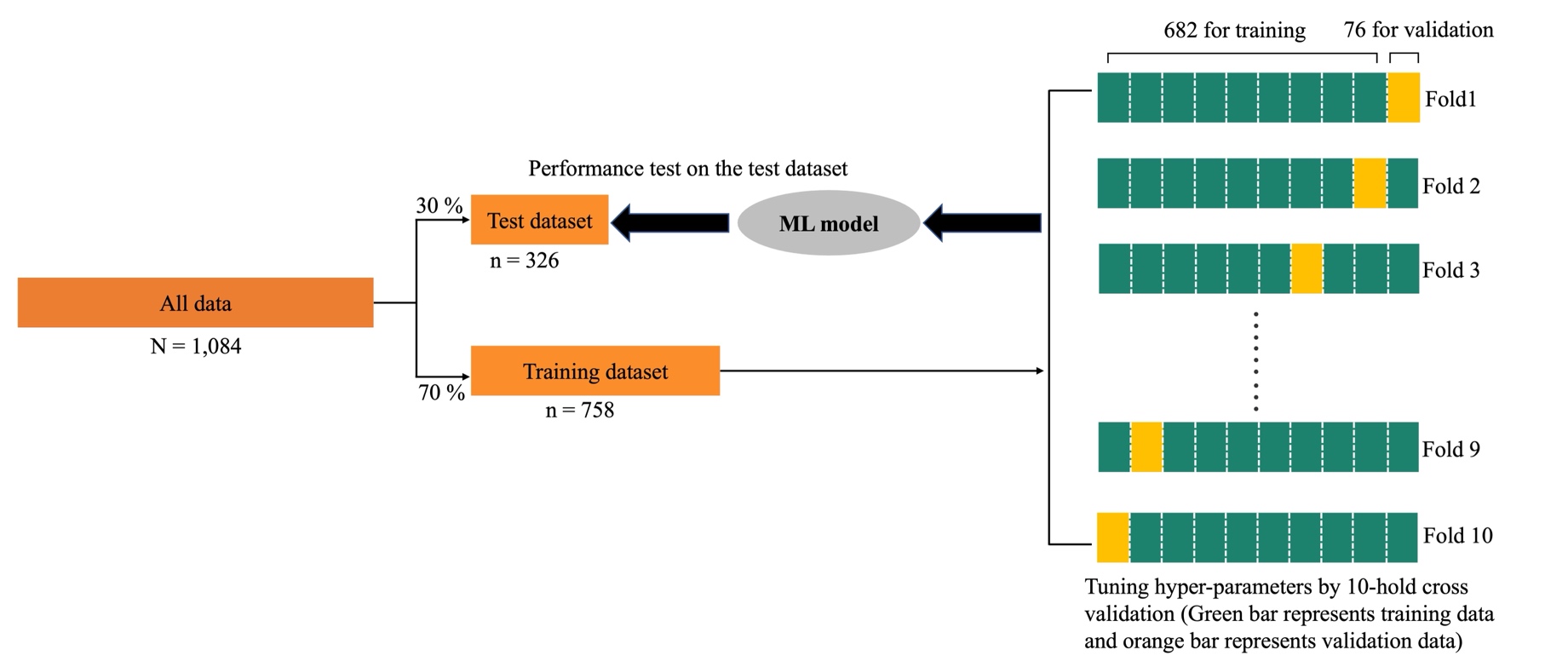


**Detailed method of developing the non-ML model**

Univariate and multivariate logistic regression analyses were performed on the training dataset to identify significant predictors of overall post-ESD bleeding among 20 pre- and peri-treatment variables, as listed in Table S1. Variables with *p* < 0.05 in the univariate analysis were included in the multivariate analysis. Subsequently, variables with *p* < 0.05 in the multivariate analysis were selected for inclusion in the non-ML model. The β regression coefficients for each significant variable were calculated from the corresponding odds ratios. Each variable that was significant in the multivariate analysis was assigned a score derived from its β regression coefficient, with the assigned score being the closest integer to the actual β regression coefficient multiplied by two, in accordance with a previous report [E2]. The total score, obtained by summing the scores of all selected variables, was used as a risk score to predict overall post-ESD bleeding. This risk score-based prediction was considered the non-ML model.

**References**

E1. Prokhorenkova L, Gusev G, Vorobev A, Dorogush AV, Gulin A. CatBoost: unbiased boosting with categorical features [Internet]. arXiv [cs.LG]. 2017. Available from: http://arxiv.org/abs/1706.09516

E2. Hatta W, Tsuji Y, Yoshio T, Kakushima N, Hoteya S, Doyama H, et al. Prediction model of bleeding after endoscopic submucosal dissection for early gastric cancer: BEST-J score. Gut. 2021;70:476–84.
