# Supplementary material for: Machine‐Learning Prediction of Bleeding After Endoscopic Submucosal Dissection for Early Gastric Cancer: A Multicenter Study
Source: JGH Open. 2025 Jun 29;9(7):e70203. doi: 10.1002/jgh3.70203 (PMC12206847; doi:10.1002/jgh3.70203)
Supplement: Supplementary file 5 — TABLE S1. Logistic regression analysis of factors predicting post‐ESD bleeding in the training data set. [file JGH3-9-e70203-s003.docx]

TABLE S1. Logistic regression analysis of factors predicting post-ESD bleeding in the training dataset

|  | **Univariate** | | **Multivariate** | | | |
| --- | --- | --- | --- | --- | --- | --- |
|  | **OR (95% CI)** | ***p* value** | **OR (95% CI)** | ***p* value** | **β regression coefficient** | **Assigned points** |
| Age ≥ 75 years | 1.62 (0.87–3.03) | 0.13 |  |  |  |  |
| Female sex, n (%) | 1.14 (0.57–2.26) | 0.71 |  |  |  |  |
| Hypertension | 1.46 (0.75–2.84) | 0.27 |  |  |  |  |
| Diabetes mellitus | 1.16 (0.54–2.46) | 0.71 |  |  |  |  |
| Atrial fibrillation | 7.24 (3.81–13.8) | <0.01 | 0.64 (0.23-1.82) | 0.41 | -0.44 |  |
| Valvular disease | 1.63 (0.66–4.01) | 0.29 |  |  |  |  |
| Ischemic heart disease | 1.70 (0.64–4.51) | 0.28 |  |  |  |  |
| Chronic heart failure | 1.22 (0.61–2.42) | 0.57 |  |  |  |  |
| Chronic renal failure on HD | 3.35 (0.71–15.8) | 0.13 |  |  |  |  |
| Liver cirrhosis | 1.01 (0.13–7.83) | 0.99 |  |  |  |  |
| DVT/PE | 0.00 (0.00–Inf) | 0.98 |  |  |  |  |
| DOAC | 7.15 (3.72–13.8) | <0.01 | 18.2 (5.78–57.2) | <0.01 | 2.90 | 6 |
| Low-dose aspirin | 0.76 (0.18–3.26) | 0.71 |  |  |  |  |
| Cilostazol | 0.00 (0.00–Inf) | 0.98 |  |  |  |  |
| P2Y12RA | 6.92 (2.74–17.5) | 0.00 | 7.69 (2.27–26.1) | <0.01 | 2.03 | 4 |
| Warfarin | 8.65 (4.15–18.1) | 0.00 | 18.8 (5.62-63.2) | <0.01 | 2.93 | 6 |
| Heparin bridge | 6.67 (3.51–12.7) | 0.00 | 1.50 (0.63-3.56) | 0.36 | 0.41 |  |
| Location, U (vs. L and M) | 0.96 (0.40–2.33) | 0.93 |  |  |  |  |
| Ulceration | 0.45 (0.11–1.88) | 0.27 |  |  |  |  |
| Tumor size > 30 (mm) | 0.75 (0.22–2.48) | 0.63 |  |  |  |  |
| Abbreviations: DOAC, direct oral anticoagulant; DVT, deep venous thrombosis; ESD, endoscopic submucosal dissection; HD, hemodialysis; OR, odds ratio; PE, pulmonary embolism; P2Y12 RA, P2Y12 receptor antagonists; SM, submucosa | | | | | | |
